# Indocyanine Green Angiography Guided Management of Vogt-Koyanagi-Harada Disease

**Published:** 2011-10

**Authors:** Nadia Bouchenaki, Carl P. Herbort

**Affiliations:** 1 Inflammatory and Retinal Eye Diseases, Center for Ophthalmic Specialized Care, Lausanne, Switzerland; 2 Mémorial A. de Rothschild Clinique Générale-Beaulieu, Geneva, Switzerland; 3 University of Lausanne, Lausanne, Switzerland

**Keywords:** Vogt-Koyanagi-Harada Disease, Indocyanine Green Angiography, Therapy

## Abstract

**Purpose:**

To report the management of Vogt-Koyanagi-Harada (VKH) disease based on indocyanine green angiography (ICGA).

**Methods:**

VKH patients with acute episodes of inflammation (inaugural or recurrent) who had received standard ICGA-guided care were studied retrospectively. Standard of care included high dose systemic corticosteroids at presentation and close ICGA follow-up with addition of immunosuppressive agents and/or intensification of ongoing therapy when recurrent choroidal lesions were detected by ICGA. Visual acuity, number of subclinical recurrences, type and duration of therapy, proportion of quiescent patients after therapy, and ICGA findings were recorded.

**Results:**

Nine patients including 8 female and one male subject were studied. Five patients had inaugural disease and 4 presented with recurrent acute episodes. Visual acuity increased from 0.86±0.36 to 1.14±0.34 in the right eyes, and from 0.77±0.34 to 1.05±0.33 in the left eyes. The number of ICGA-detected occult choroidal recurrences amounted to 13. Mean duration of treatment was 30.1±34.6 months leading to recurrence-free status after discontinuation of therapy in 6 cases with mean duration of 29.5 months.

**Conclusion:**

Continuous monitoring and aggressive therapy guided by ICGA in VKH disease prolongs treatment as compared to textbook guidelines but offers the prospect of reaching inflammation-free status after discontinuation of therapy. Zero tolerance to subclinical choroidal inflammation avoids irremediable evolution towards sunset glow fundus in patients treated early after the initial acute inflammatory attack.

## INTRODUCTION

Vogt-Koyanagi-Harada (VKH) disease is a bilateral granulomatous choroiditis that invariably starts in the choroidal stroma and is therefore called a primary stromal choroiditis.^1^ The inflammatory process may evolve silently in the choroid without being clinically detected. When the condition becomes clinically apparent, it initially presents as panuveitis or more frequently as a predominantly posterior uveitis with papillitis and exudative retinal detachments. The disease is an autoimmune T-helper 1 type reaction directed against melanocytes in the choroidal stroma which explains its site of origin.^2^,^3^

The inflammatory reaction in VKH can sometimes be mild and remain subclinical, making its diagnosis difficult without sensitive investigational tests. Most of the time, however, the inflammatory reaction is hyperacute involving the retina, optic nerve, vitreous and anterior chamber. Although these structures are involved only as a consequence of primary choroidal inflammation, they are accessible to clinical examination and will render the disease clinically apparent by demonstrating classical signs of multiple exudative retinal detachments and papillitis. These clinical signs respond to inflammation suppressive therapy (IST) including high dose systemic corticosteroids, which is necessary for control of hyperacute disease.^4^–^6^ After initial aggressive IST, clinical disease is rapidly brought under control within 6 to 16 weeks and treatment is then progressively tapered with a total treatment duration of 6 to 9 months according to textbooks.^7^,^8^

Development of sunset glow fundus is reported in almost 100% of VKH cases, even when the disease seems to be under control clinically.^9^–^11^ Development of sunset glow fundus in the absence of clinically manifest disease suggests silently progressive choroidal inflammation and indicates that the post-acute treatment regimen may be insufficient.^12^

Before the development of indocyanine green angiography (ICGA), there was no sensitive method to detect subtle choroidal inflammation, and ultrasonography was the only imaging modality used for VKH disease by providing only gross information in pronounced disease. In contrast to ultrasonography, ICGA is sensitive enough to detect small inflammatory foci within the choroidal stroma as well as inflammation of choroidal stromal vessels.^13^ ICGA is currently the most appropriate method for investigating choroidal inflammatory diseases, as it is sensitive enough to demonstrate minimal and often, subclinical lesions of the choroid both in inaugural disease and in insufficiently treated disease.^14^–^17^ ICGA signs of acute VKH disease have been clearly standardized with four signs consistently present. These include early choroidal vessel hyperfluorescence, intermediate to late phase fuzziness of choroidal stromal vessels, disc hyperfluorescence, and hypofluorescent dark dots (HDDs).^16^ The contribution of ICGA is particularly useful in cases of suspected VKH disease without an acute onset which are usually seen at a later stage, when suboptimal therapy has already been initiated, as well as in cases without a complete set of signs in whom diagnosis is more difficult. In such cases, ICGA signs indicating choroidal inflammatory activity include HDDs and fuzziness of stromal vessels; the other two signs (early hyperfluorescent choroidal vessels and disc hyperfluorescence) are indicative of more acute disease.^18^–^22^

Choroidal disease in VKH is silently progressive; therefore to avoid late complications such as sunset glow fundus, the inflammation needs to be treated adequately. On the other hand, only ICGA monitoring allows detection of subclinical inflammation and correct orientation of treatment. Herein we report the results of ICGA-guided management of acute episodes (inaugural or recurrent) of VKH disease.

## METHODS

Patients with a diagnosis of VKH disease in an acute stage (inaugural episode or acute recurrent disease) who were referred to the uveitis clinic of the Center for Ophthalmic Specialized Care (COS) from 1995 to 2010 were retrospectively studied.

All patients had a complete routine uveitis work-up as applied for patients with posterior uveitis. At each major visit a complete ocular examination was performed including Snellen visual acuity, slit lamp examination, applanation tonometry, dilated fundus examination, laser flare photometry, computerized visual field (VF) testing, optical coherence tomography (OCT) since available, and dual fluorescein angiography (FA) and ICGA. FA and ICGA were performed using a standard protocol as previously described.^23^ FA and ICGA were usually performed simultaneously. A Topcon 50 IA camera (Topcon, Tokyo, Japan) coupled to the ImageNet (Topcon, Tokyo, Japan) image digitalizing system was used to acquire the images.

To exclude autofluorescence, evidence of pre-injection fluorescence was sought with the highest flash intensity used for ICGA. At the same time, red-free posterior pole frames were taken. After a bolus injection of 500 mg ICG (Cardiogreen; Peaselt, Lorei, Germany) diluted in 7.5 ml of 0.9% salt solution, ICGA frames of the posterior pole were taken up to 2 to 3 minutes (early phase angiogram). ICG background fluorescence was analyzed in detail 12±3 minutes after ICG injection (intermediate phase angiogram) by capturing posterior pole frames and a minimum of 8 frames from the whole periphery over 360°. At the end of the intermediate ICGA phase, FA was performed. Early fluorescein angiographic frames of the posterior pole were taken up to 2 minutes. Between 4 to 7 minutes, 360° panoramic imaging of the periphery was performed (minimum of 8 frames), followed by late fluorescein posterior pole frames at 10 minutes. Late ICG background fluorescence was analyzed at 32±4 minutes after ICG injection (late phase angiogram) in the same fashion as that during the intermediate phase.

Laser flare assessment was performed using a Kowa FM-500 laser flare meter (Kowa Company, Ltd., Electronics and Optics Division, Tokyo, Japan) and OCT was performed using the OTI-Spectral OCT/SLO (OTI Inc., Toronto, Canada). For VF assessment the G1 program of the OCTOPUS 900 (Octopus 900, G Standard; Haag-Streit International, Bern, Switzerland) was used.

**Figure 1:**
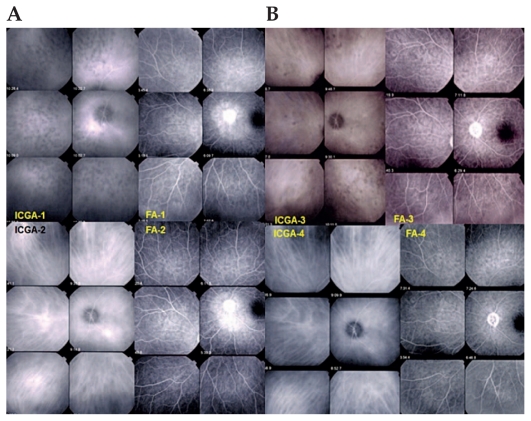
**(A)** An example of ICGA-guided management of VKH. A patient with an inaugural episode of VKH disease shows numerous HDDs and complete vessel fuzziness (top left frames, ICGA-1). FA (top right frames, FA-1) shows disc hyperfluorescence and peripapillary exudative retinal detachment. After intravenous methylprednisolone and oral prednisone, there is complete disappearance of HDDs and fuzziness of choroidal vessels, the pattern of which is clearly seen again (bottom left frames, ICGA-2), while there is residual disc and peripapillary hyperfluorescence (bottom right frames, FA-2). **(B)** After prednisone tapering, subclinical recurrence of choroidal inflammation with reappearance of HDDs and fuzziness of choroidal vessels (top left frames, ICGA-3) are seen, while FA is quasi normal with only residual disc hyperfluorescence (top right frames, FA-3). After increase of oral prednisone and addition of mycophenolate mofetil, the choroidal inflammatory lesions (HDDs) disappeared two months later and choroidal vessels regained a normal appearance (bottom left frames, ICGA-4). ICGA, indocyanine green angiography VKH, Vogt-Koyanagi-Harada HDDs, hypofluorescent dark dots FA, fluorescein angiography

All patients underwent the standard of care of our center (Fig. 1) for treatment of acute active episodes of VKH disease (inaugural disease or acute recurrences) with very high dose oral corticosteroids (prednisone, 1.2–1.7 mg/kg) or in case of hyperacute disease, a combination of intravenous methylprednisolone (1000 mg/day) for 3 days followed by high-dose oral corticosteroids (prednisone, 1 mg/kg). ICGA, according to the above mentioned protocol, was performed at presentation before initiating treatment and every month±1 week thereafter until disappearance of HDDs and normalization of choroidal vessels, at least up to 4 months after presentation. During the tapering phase of corticosteroids beyond 4 months, ICGA was performed every 6±2 weeks. In case of subclinical reappearance of HDDs and/or fuzzy choroidal vessels, corticosteroid therapy was increased to 1mg/kg again and in parallel an immunosuppressant agent was added, azathioprine or mycophenolate mofetil usually being the first choice. Progressive tapering was then started again under ICGA control. Therapy was increased again by two steps each time ICGA signs reappeared.

Visual outcomes, proportion of cases having presented with subclinical recurrence, proportion of cases having needed additional immunosuppressive therapy, type and duration of therapy, quiescence of disease at the end of follow-up, course of fundus appearance, and presence and evolution of ICGA signs (ICGA sign free cases) were the main parameters analyzed in the study.

## RESULTS

Out of 1,268 new patients seen at the uveitis clinic at the Center for Ophthalmic Specialized Care (COS), Lausanne, Switzerland from 1995 to 2010, 22 (1.73%) subjects including 15 women and 7 men were diagnosed with VKH disease. Nine patients (1 man and 8 women) with mean age of 33.9±10.6 years had sufficient data to be included in the present study (Table 1). One patient had ICGA-guided follow-up who responded insufficiently to several treatments probably because of lack of compliance; this case was excluded from final analysis.

**Figure 2:**
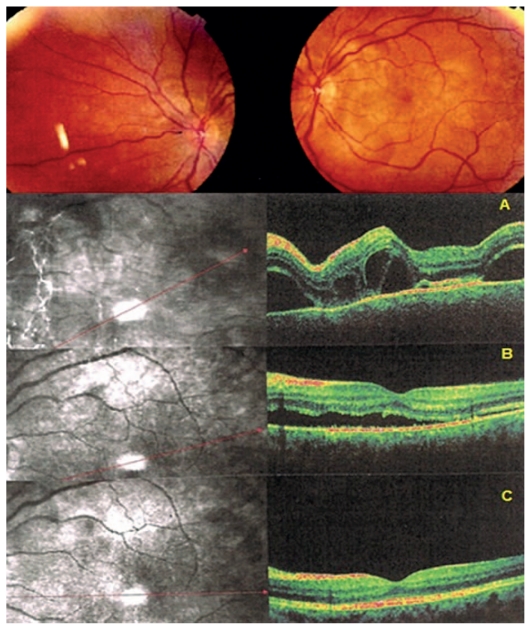
Fundus and OCT findings in acute VKH. Fundus signs showed bilateral serous retinal detachments in this patient (only the left eye is shown) diagnosed 7 days after the onset of symptoms. OCT illustrates subretinal fluid at presentation (top scan, A). After 3 days of intravenous methylprednisolone, the amount of fluid has decreased substantially (middle scan, B) with quasi normalization after one month of treatment (bottom scan, C). OCT, optical coherence tomography VKH, Vogt-Koyanagi-Harada

Five subjects were seen for an acute inaugural episode, 3 cases experienced an acute recurrence and had been initially treated at another center, and the ninth patient had uncontrollable disease. Mean duration from onset of symptoms (initial episode or acute recurrent attack) to initiation of therapy was 19.1±13.1 days (excluding the ninth patient). Serous retinal detachment was seen in 7 of 9 cases (Fig. 2); 8 of 9 patients had cerebrospinal fluid (CSF) pleocytosis. The patient with normal CSF was diagnosed 7 days after the onset of symptoms.

Mean visual acuity at presentation was 0.86±0.36 and 0.77±0.34 in the right and left eyes respectively, and 1.14±0.34 and 1.05±0.33 respectively at final follow-up.

The mean number of ICGA recurrences was 2.1±1.4 episodes with 3 patients demonstrating no clinical or ICGA signs of recurrence after the initial high-dose treatment was tapered. Excluding these 3 patients, the mean number of recurrences was 3.2±1.7 in the remaining 6 patients. The total number of ICGA recurrences was 19, with 6 recurrences (all in one poorly responsive case) being both clinical and ICGA-based.

All patients were treated using high-dose corticosteroid therapy, this included high-dose intravenous methylprednisolone (500–1000 mg daily) for 3 days in 4 of 9 patients, and oral prednisone (1.2–1.7 mg/kg) in the remaining. In 2 of 9 (22%) patients no immunosuppressive agent was added and no recurrence occurred after slow tapering of steroids. In 7 of 9 patients (78%) however, immunosuppressive therapy was required in addition to prednisone therapy from the beginning. In one patient, azathioprine was added after one month because of corticosteroid intolerance and slow response to therapy based on ICGA findings. This patient experienced no recurrence after tapering treatment. In the 6 remaining patients immunosuppressive agents had to be added because of recurrences during tapering of initial treatment. These included azathioprine (N=5), mycophenolate mofetil (N=4), cyclosporine (N=3), and infliximab, adalimumab and interferon-α (N=1 for each). Two patients required simultaneous use of two immunosuppressive agents and three patients received two immunosuppressants sequentially, i.e. azathioprine was replaced by the less toxic mycophenolate mofetil.

Mean total duration of therapy was 30.1±34.6 (range, 9–114) months. In the patient resistant to therapy, treatment was administered continuously for 132 months. Six patients remained quiescent, developed no ICGA signs and were recurrence-free for a mean duration of 29.5±22.7 months after cessation of treatment. One patient was lost to follow-up while still under treatment and recurrence-free for 6 months and the other patient was still under treatment at the time of this report, 13 months after the acute episode.

Sunset glow fundus sign was present in 4 of 9 patients, all of whom had experienced recurrences including the poorly responsive patient. In 5 other patients there were no sunset glow fundus signs, all of whom were first seen at the time of their inaugural acute episode. None of the patients showed late signs of disease such as poliosis, alopecia or vitiligo.

**Figure 3:**
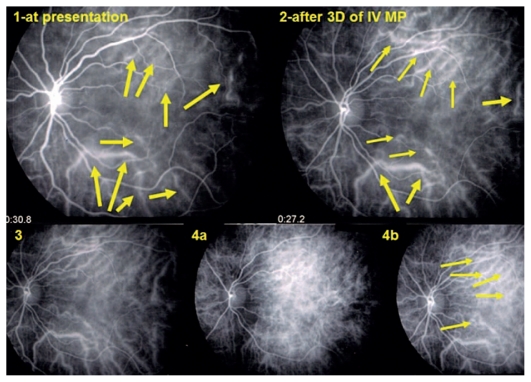
Early choroidal hyperfluorescent vessels three days after administration of intravenous methylprednisolone (top right) in a patient with an acute inaugural VKH episode at presentation (top left). Normalization of choroidal vessels after 3 months of combined prednisone and mycophenolate mofetil therapy (bottom left, 3). Reappearance of early hyperfluorescent choroidal vessels, together with HDDs 6 weeks later upon discontinuation of prednisone (bottom frames 4a and 4b). VKH, Vogt-Koyanagi-Harada HDDs, hypofluorescent dark dots

**Figure 4:**
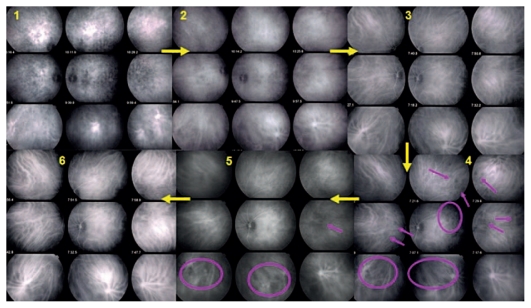
Numerous HDDs and fuzzy vessels at presentation (1). Decrease in HDDs after one month of prednisone therapy with persistence of fuzzy vessels observed with ICGA-guided follow-up (2). Choroidal inflammation finally responded to combined prednisone and mycophenolate mofetil therapy resulting in resolution of HDDs and restoration of normal pattern of choroidal vessels (3). Six weeks later and after discontinuation of prednisone, recurrence of occult choroidal inflammation was noted with reappearance of HDDs and vessel fuzziness in some areas of the fundus (4, crimson circles and arrows) which slowly responded to the addition of cyclosporine after two months (5) leading to a quasi normal choroidal appearance after another two months (6). ICGA, indocyanine green angiography HDDs, hypofluorescent dark dots

ICGA signs were identical whether the acute episode was inaugural or recurrent. At presentation, 8 of 9 patients showed early hyperfluorescent choroidal vessels (Fig. 1), 8 of 9 subjects presented with disc hyperfluorescence which was unilateral in two, all cases showed fuzzy choroidal vessels bilaterally (Fig. 3) and bilateral round HDDs (Fig. 4). At the time of occult choroidal recurrence, HDDs and fuzziness of vessels were the only ICGA signs.

## DISCUSSION

The initial pathological process in VKH disease starts within the choroidal stroma at the level of melanocytic islets and goes clinically undetected until inflammation affects neighboring structures including the retina and optic disc, usually in an acute or hyperacute fashion.^24^–^26^ The standard of care is to start vigorous IST which classically consists of high-dose systemic corticosteroids tapered over a period of 6–9 months.^7^,^8^ The disease responds well to such an approach and clinical signs regress satisfactorily.^27^ Nevertheless, even in patients with apparently inactive disease, close to 100% of cases evolve towards sunset glow fundus.^10^,^28^ This evolution towards depigmentation of the fundus despite apparently quiescent disease shows that persistent choroidal disease, albeit without clinical signs, is invariably present after clinical disease has been brought under control.^29^,^30^ Subclinical choroidal inflammation in posterior uveitides can only be detected by ICGA.^31^–^38^ This is the case for VKH at the onset of disease when no clinical signs are present yet, or after treatment once clinical disease has been mastered.^14^,^22^ It has been shown that in seemingly pure anterior recurrences of VKH, ICGA clearly shows subclinical choroidal lesions.^17^ We have also shown that insufficient therapy in the post-acute phase when clinical signs have resolved, fails to control choroidal lesions.^15^ The amount of evidence at our disposal on continuing smouldering choroidal inflammation clearly indicates that in order to extinguish VKH disease, subclinical choroidal inflammation has to be treated and the only way to assess and follow choroidal involvement is by using ICGA.

ICGA-guided therapy has been our standard of care since we used ICGA for posterior uveitis back in the early 90′s. The current study showed that in 8 patients, a number of 13 subclinical recurrences were detected by ICGA leading to reinforced and prolonged therapy. Mean duration of treatment in our series was 30 months which is by far longer than indicated in textbooks.^7^,^8^ However the absence of sunset glow fundus in patients who had received ICGA-guided therapy since their inaugural episode justifies such an aggressive therapeutic approach. In contrast, all patients included for an acute recurrence developed sunset glow fundus (sometimes already present at inclusion) most certainly due to previous damage caused by ongoing smouldering subclinical choroidal inflammation. Furthermore, visual outcome was excellent for this group of VKH patients receiving ICGA-guided therapy. Such an aggressive and closely monitored attitude is further supported by the good proportion of patients in whom the disease remained quiescent after discontinuation of therapy with mean inflammation-free status exceeding two and a half years. This group of patients with quiescent disease included most of the patients in whom the acute episode had been a recurrence and sunset glow fundus was present. None of our patients developed late signs of VKH disease such as poliosis, vitiligo or alopecia indicating that zero tolerance even to low grade choroidal inflammation prevents evolution to late stage disease including sunset glow fundus in patients taken care of since the inaugural inflammatory episode. We believe that classification criteria^39^ employing late signs are obsolete because they do not characterize late disease, in fact they reflect insufficiently treated disease.

ICGA signs have been standardized for early acute VKH disease and findings were found to be quite consistent.^16^ Four main ICGA signs have been suggested to assess choroidal involvement in acute episodes of VKH. These include (1) early hyperfluorescent choroidal vessels indicating acute choroidal vasculitis, (2) disc hyperfluorescence reflecting hyperacute disease, (3) fuzzy choroidal vessels seen in intermediate to early late phase frames denoting choroidal inflammatory vasculopathy, and (4) HDDs indicating foci of choroidal inflammation. All of these signs were present in our group of patients with acute inflammatory attacks, except in one patient who did not demonstrate disc hyperfluorescence and another patient who did not show early hyperfluorescent choroidal vessels. At the time of occult choroidal recurrence, the degree of inflammation is much less severe than the initial acute episode so that disc hyperfluorescence is never observed and early hyperfluorescent choroidal vessels were either absent or not clear-cut. All 13 episodes of occult recurrence were clearly identified by the reappearance of HDDs in parallel with fuzziness of choroidal vessels.

To meaningfully assess posterior uveitis nowadays, it is difficult to imagine not using ICGA when choroidal involvement is suspected. ICGA represents the only modality capable of identifying choroidal inflammation, hidden to other investigational modalities. This peculiarity of choroidal inflammation is characterized by the analogic angiographic concepts including the “iceberg effect” where most of the inflammatory process is not detectable by usual diagnostic means, and the “submarine effect” when only ICGA is positive and no other clue to choroidal inflammation exists. Obviously if choroidal inflammation can only be assessed by ICGA, monitoring of its evolution and adjustment of therapy can also only be performed by ICGA follow-up, as was illustrated in this study.

## Figures and Tables

**Table 1:** Demographic data, type of disease, follow-up period and ICGA findings

Cases (Sex)	Age (Years)	Type of acute episode	F/U (months)	ICGA signs			
				Early hyperfluorescent vessels	Disc hyperfluorescence	Fuzzy vessels	HDDs
Case 1 (F)	37	Inaugural	Under therapy	Yes	No	Yes	Yes
Case 2 (F)	19	Recurrent	4 m	Yes	Yes	Yes	Yes
Case 3 (F)	31	Inaugural	Lost to F/U	Yes	Yes	Yes	Yes
Case 4 (F)	35	Inaugural	14 m	Yes	Yes	Yes	Yes
Case 5 (F)	24	Recurrent	114 m	Yes	Yes	Yes	Yes
Case 6 (M)	46	Inaugural	9 m	Yes	Yes	Yes	Yes
Case 7 (F)	51	Recurrent	28 m	Yes	Yes	Yes	Yes
Case 8 (F)	24	Inaugural	14 m	No	Unilateral	Yes	Yes
Case 9 (F)	38	Recurrent	132 m	Yes	Unilateral	Yes	Yes

F, Female; M, Male; ICGA, indocyanine green angiography; F/U, follow-up; HDDs, hypofluorescent dark dots
